# Four Cases of Parturient Apoplexy1Presented to the semi-annual meeting of the Pennsylvania State Veterinary Medical Association, Franklin, September 21, 1897.

**Published:** 1897-10

**Authors:** W. E. McCray

**Affiliations:** Oil City, Pa.


### FOUR CASES OF PARTURIENT APOPLEXY.[1]

By W. E. McCray, V.S.,
Oil City, Pa.

I desire to call your attention to four cases of parturient apoplexy that I have attended within the last six months. As you are all familiar with the pathology and symptoms of this disease, I will not enter into details.

Case I.—A thoroughbred Jersey cow had her calf about 8 o'clock one evening, and went down about 3 o'clock the following morning. This was a well-marked case, with all the symptoms prominent; paralysis of the posterior extremities and contraction of the muscles of the cervical region, with head crooked tight to flank, emitting a grunt at every respiration.

The following line of treatment was adopted: A purgative was administered, a mustard-plaster applied to the back, hot water injections, and ice applied to the head. Stimulants were given every two hours. About 5 o'clock in the evening of the same day I was called again. The animal was undoubtedly no better, and coma more pronounced. I gave her one grain of eserine sulphate, intravenous injection, and applied the pump. Inside of twenty minutes the tension of the cervical muscles relaxed, and she showed prominent symptoms of the physiological actions of

[1] Presented to the semi-annual meeting of the Pennsylvania State Veterinary Medical Association, Franklin, September 21, 1897.

this drug. She had two evacuations of hard feces, and then seemed to rest easier with head erect. The following morning I gave her one-half grain more of the drug, and by noon she was upon her feet. The subordinate treatment consisted of stimulants with nux vomica in small doses.

CASE II.—A grade Durham that went down sometime the second day after calving. Was seen by me about six hours after going down. I at once administered one-and-a-quarter grains of eserine, and applied the pump. She responded to the action of the drug. I also gave her a purgative, and ordered stimulants every hour. She was on her feet in six hours, and eventually made a good recovery.

CASE III.—An old grade Jersey. Had gone down the third day after calving, and had been down twenty-four hours when seen by me. The owner said he had given her "two quarts of crude oil and two pounds of salts." I gave her treatment the same as in other cases, and she was upon her feet the next morning and lived six days. She then died with mechanical pneumonia, due to the administration of the oil and salts.

CASE IV.—A high-bred Jersey. Had her calf in the morning I was called the same evening, the owner stating that "something ailed the cow." She was down by the time I got to the field. I immediately gave her treatment as heretofore mentioned. That night she lay in the rain. I called the next morning, gave her the second injection, and hauled her to the barn on a stone-boat. About 11 o'clock that morning she rose to her feet, gained strength during the day, and when I made my last visit that evening she was up and the calf was sucking.

Now, gentlemen, in bringing these cases to your notice I do not wish to be understood as advancing any new ideas, but simply to give you my experience in the action of this drug in the treatment of parturient apoplexy.

